# Supplementary material for: Relative importance of socioecological domains to predicting opioid-involved mortality
Source: PLoS One. 2025 Jul 29;20(7):e0328286. doi: 10.1371/journal.pone.0328286 (PMC12306787; doi:10.1371/journal.pone.0328286)
Supplement: S1 Supporting Methodology — (DOCX) [file pone.0328286.s001.docx]

# Supporting Information

**S1 Table 1:** Potency Ranking by Milligrams Morphine Equivalent

| **Drug** | **Rank** |
| --- | --- |
| Fentanyl & Analogs | 1 |
| Levorphanol | 2 |
| Hydromorphone | 3 |
| Heroin | 4 |
| Methadone |  |
| Oxymorphone |  |
| Oxycodone | 5 |
| Hydrocodone | 6 |
| Morphine |  |
| Codeine | 7 |
| Meperidine | 8 |
| Tramadol |  |

**S1 Table 2:** Factors Organized by Socioecological Domain

| **Socioecological Domain** | **Factor** | **Source** | **Description** |
| --- | --- | --- | --- |
| Individual Non-Drug | -ICD Diagnosis Codes  -Days of care  -Type of admission  -Days of care in ICU; inpatient only  -Flag if encounter is an inpatient encounter  -Expected source of payments  -Age  -Sex | NHCS | These variables related to individuals’ characteristics or personal care experience, but were not involving drugs encountered or drug-related conditions. |
| Individual Drug | -ICD Diagnosis Codes  - Specific opioids involved in the hospital encounter | NHCS enhanced opioid-identification file | These variables related to individuals’ encountered drugs or drug-related conditions. |
| Hospital | -Number of beds at the hospital  -Hospital census division  -Hospital ownership status  -Service type of hospital  -Point of origin  - Urban/Rural designation | NHCS | Hospital-level factors that would influence the overall level of care, resources available, and expertise of providers. |
| Community | -Food environment index  -Uninsured percentage  -High school graduation percentage  -Unemployment percentage  -Children in poverty percentage  -Income ratio between 80th and 20th percentiles  -Violent crime population rate  -Households experiencing severe housing problems percentage  -Experiencing food insecurity percentage  -Median household income  -Residential segregation index – non-white/white  -Not proficient in English percentage  -Population rural percentage  -Fair or poor health percentage  -Number of poor physical health days  -Number of poor mental health days  -Adult smoking percentage  -Physically inactive percentage  -Access to exercise opportunity percentage  -Excessive drinking percentage  -Primary care physician population rate  -Mental health providers population rate  -Preventable hospital stay population rate  -Frequent physical distress percentage  -Frequent mental distress percentage  -Total dispensing of buprenorphine, codeine, fentanyl, hydrocodone, hydromorphone, levorphanol, meperidine, methadone, morphine, oxycodone, oxymorphone, tramadol | Robert Wood Johnson Foundation County Health Rankings  IQVIA Longitudinal Patient Database | Community factors encompassed indicators for the built environment (e.g., food insecurity) and affluence (e.g., median income), health metrics (e.g., providers per population), and the overall prescription drug dispensing in a community |
