# Supplementary material for: Relative importance of socioecological domains to predicting opioid-involved mortality
Source: PLoS One. 2025 Jul 29;20(7):e0328286. doi: 10.1371/journal.pone.0328286 (PMC12306787; doi:10.1371/journal.pone.0328286)
Supplement: Attachment — Submitted filename: Review of PLOS ONE MS PONE-D-24-46562.docx [file pone.0328286.s002.docx]

**Review of PLOS ONE MS #** **PONE-D-24-46562**

**Relative Importance of Socioecological Domains to Predicting Opioid Overdose**

1. Is the manuscript technically sound, and do the data support the conclusions? The manuscript must describe a technically sound piece of scientific research with data that supports the conclusions. Experiments must have been conducted rigorously, with appropriate controls, replication, and sample sizes. The conclusions must be drawn appropriately based on the data presented.

The term “opioid deaths originating in hospitals” not well-defined nor well-measured in the study:

- People who receive an opioid during a hospital encounter could reflect acute treatment, end of life treatment, or chronic use – all of which do not necessarily lead to death
- We cannot be certain that the death “originated” in the hospital given that the authors define this as individuals “who died in 2016-2017 (primary outcome) during or *subsequent* to the hospital encounter.” How soon after the hospital encounter is the “subsequent” death? I would restrict the study to deaths occurring in the hospital.

The way “opioid involved death” is interpreted in this study is somewhat misleading given that the motivation of the study is to address the opioid crisis:

- Although the Drug-Involved-Mortality supplements to the National Death Index are relatively reliable for identifying cause-of-death involving opioids, according to the CDC, involvement includes therapeutic use (e.g., end of life) or simply “being on board” which would have a very different interpretation than an overdose. Moreover, the “progression” from lower to high intensity opioid could reflect the standard of care for treatment as the patient nears death (e.g., for cancer) rather than an overdose, creating a mechanical relationship between opioid involved mortality and progression in intensity. I would drop the “progression” outcome from the analysis entirely.
- From <https://www.cdc.gov/rdc/b1datatype/datafiles/Drug-Involved-Mortality-Data-Documentation.pdf> : “Involvement encompasses everything a substance can do or be used for. This includes overdose (poisoning); adverse reactions; being present on board; therapeutic use; being used, misused, or abused; exposure not otherwise specified; complicating health conditions; interacting with other substances; and causing drug dependence or being used by drug dependent persons. Involvement may occur at or prior to time of death (e.g., “HISTORY OF HEROIN ABUSE”). The underlying assumption is that the substances mentioned on the death certificate contributed to death. However, this assumption may not be true in some cases. For example, a substance mentioned as being present on board may have not contributed to death. In addition, a substance could have been used to treat the health conditions that led to death, but also did not contribute to death. Lastly, the death certificate literal text may not contain sufficient information to ascertain exactly how a substance was involved in death. For example, the literal text may simply mention “HEROIN”, with no contextual information on whether heroin resulted in overdose or whether the decedent had a history of heroin abuse. These cases are also assumed to be substance-involved deaths.

The sample of hospitals in the study does not seem representative.

- The study includes a total of 103 hospitals out of the 7,341 that operate nationwide. Although the study states the sample of hospitals is a mix of rurality, service type, size, ownership, and geographic regions, the question is relative to what? None of the hospitals in the sample are in the Northeast, compared to 14.3% of hospitals nationwide. I would encourage the authors to obtain national statistics on each hospital characteristic to benchmark their sample for the reader rather than just stating it’s not nationally representative.

The results obtained from the population studied do not seem generalizable to understanding the opioid overdose crisis.

- Of the 429,005 patients with opioid-related encounters that were analyzed, more than half (56.6%) were female. This was true with respect to every drug type except heroin, which stands in contrast to CDC data showing that of the 27.9 million ED visits related to drug use in the DAWN database, visits by men were 2.69 times more likely to involve illicit drugs than visits by women (<https://pmc.ncbi.nlm.nih.gov/articles/PMC4899061/> ). This suggests to me that the population in this study are patients seeking care for other health-related conditions rather than “fatal opioid overdoses that occurred after hospital encounters involving opioids.”
- Also, while I appreciate that the authors acknowledge that the primary limitation of the study is that data are from 2016-2017, prior to the substantial rise in fentanyl-involved overdose deaths, I do not agree with their assessment that “the relative impact of different factors is potentially unchanged since these data were collected.” Given the swift rise in fentanyl use, misuse and overdose deaths, it is likely or at least plausible that fentanyl now swamps other community factors in terms of relative importance.

The results in Table 3 regarding the relative contribution of the many factors listed are neither novel nor surprising.

- The results in Table 3 do not seem as important as the results summarized in the figure in the supplementary materials. The relative contributions of individual factors are tiny and not all that different from each other in many instances. For example, the top 3 community factors contributing to non-drug involved mortality are: 1. Excessive drinking (0.53%), 2. Household

experiencing housing problems (0.53%), and 3. Not proficient in English (0.51%).

- In other cases, the factors are quite obvious in terms of their importance. For example, in the non-drug involved mortality model, the most important individual factor was neoplastic-related diagnostic codes (e.g. cancer). The top two individual drug-related factors for drug-involved mortality were: 1. Opioid-related disorder ICD (6.2%) and 2. Poisoning by heroin ICD (2.4%). Ditto for the top factors related to progressing to higher potency: 1. Fentanyl encounter (15.5%), 2. Morphine encounter (7.3%), and 3. Hydromorphone encounter (5.5%) I am not sure what is gained by this knowledge as clinicians likely already know that OUD is the top factor leading to drug-involved mortality and that encounters involving fentanyl, morphine, and hydromorphone are likely to lead to deaths involving more potent drugs.

I could not find any table in the paper or supplementary materials that showed this result:

- “Neoplastic diagnostic codes, age, and cardiovascular diagnostic codes accounted for 150 nearly 45% of the importance in model prediction of non-drug related death.”

2. Has the statistical analysis been performed appropriately and rigorously?

One of the major limitations of using a Random Forest ML model is that of correlation bias. Out-Of-Bag prediction accuracy can overestimate the true prediction error when there are a large number of predictor variables or even small correlations between predictors. I would suggest that the study be guided more by the theory of which variables should be included as factors and how they should be measured relative to other factors rather than take the “kitchen sink” approach and include every variable that is available.

- If features are highly correlated, the importance scores might not accurately reflect the true individual contribution of each variable. There are a number of instances where the features in the study are highly correlated:
  - The study uses total prescription drug dispensing from the IQVIA Longitudinal Patient Database rather than per capita drug dispensing. Total drug dispensing is likely to be highly correlated with population, meaning there will be a high correlation between the IQVIA variables and the population density variables (e.g., rural population percentage, hospital urban/rural designation).
  - Economic variables used in this study (e.g., Unemployment percentage, Children in poverty percentage, Median household income) are all highly correlated with one another as well as other community variables related to socio-economic status (e.g., high school graduation percentage, Violent crime population rate, Households experiencing severe housing problems percentage, Experiencing food insecurity percentage, food environment index).
  - The health status variables (e.g., Fair or poor health percentage, Number of poor physical health days, Number of poor mental health days, Frequent physical distress percentage, Frequent mental distress percentage, Preventable hospital stay rate) are all highly correlated with each other and with the health behavior variables (e.g., Adult smoking percentage, Physically inactive percentage, Access to exercise opportunity percentage, Excessive drinking percentage).

The study also suffers from omitted variable bias, particularly the lack of demographic characteristics and health care delivery system variables that have been shown to affect opioid prescribing. I would encourage the authors to include these county level variables that are available from the Area Health Resource File:

- While the study includes age and gender, there are large disparities in opioid prescribing and overdose deaths across communities based on the non-white population rate and the veteran population rate.
- Similarly, while the study includes the number of providers for certain specialties (e.g., Primary care physician population rate, Mental health providers population rate), it fails to capture other important factors such as the availability of skilled nursing facilities per population and hospital beds per population, that can affect the likelihood of access to treatment.

Overall, while the Random Forest method may seem appealing in working with such a large dataset with many factors, it is also limiting in its ability to tell us anything about the magnitude of the effect. I would encourage the authors to use an Oaxaca decomposition to be able to capture both the absolute magnitude and relative contribution of each factor in predicting mortality.

- Otherwise, I worry that the study leads to false conclusions such as this, where the authors assume that community factors are more important but might be picking up on the many inter-correlated factors in that particular grouping: “Although no specific community factor contributed more than approximately 1% of importance, the combined effect was more important than individual-level factors in predicting opioid-involved overdose. This could suggest that community factors have a cumulative effect, and efforts to broadly improve the general health of communities may reduce drug overdose rates as much as drug-specific interventions.”

3. Have the authors made all data underlying the findings in their manuscript fully available? The PLOS Data policy requires authors to make all data underlying the findings described in their manuscript fully available without restriction, with rare exception (please refer to the Data Availability Statement in the manuscript PDF file). The data should be provided as part of the manuscript or its supporting information, or deposited to a public repository. For example, in addition to summary statistics, the data points behind means, medians and variance measures should be available. If there are restrictions on publicly sharing data—e.g. participant privacy or use of data from a third party—those must be specified.

Restricted data are available through the CDC Research Data Center (RDC). Access

can be obtained through submission of proposals to the RDC.

4. Is the manuscript presented in an intelligible fashion and written in standard English?

PLOS ONE does not copyedit accepted manuscripts, so the language in submitted articles must be clear, correct, and unambiguous. Any typographical or grammatical errors should be corrected at revision, so please note any specific errors here.

Specific errors:

Line 55: missing “and”

“sociodemographic, **and** past drug use history from among a wide variety of potential predictors (9-11) as”

Line 56: no capitalization of “They”

“important personal factors, though **they** have been limited to primarily including only personal domain”
